# A lunar sample renaissance

**DOI:** 10.1038/s41467-021-27296-3

**Published:** 2021-12-14

**Authors:** Tabb C. Prissel, Kelsey B. Prissel

**Affiliations:** Jacobs-JETS, Astromaterials Research and Exploration Science Division, NASA Johnson Space Center, Houston, TX USA

**Keywords:** Petrology, Planetary science, Geochemistry

## Abstract

Though the lunar samples returned by the Apollo and Luna missions have been studied for more than 50 years, scientists are discovering new clues into the early evolution of the Moon by looking through the lens of modern analytical techniques.

From the concept of global magma oceans^1,2^ to the discovery of water within a “bone-dry” Moon^3,4^, much of our geochemical understanding of planetary evolution can be attributed to the exhaustive efforts of NASA’s Apollo (1969–1972) and the Soviet Union’s Luna (1970–1976) sample return missions.

What is the role of volatiles during planetary formation and evolution? How have impact processes shaped our solar system? Long-standing questions in planetary science beckon a return to the Moon to collect new samples from previously unexplored regions. The Chinese Lunar Exploration Program has answered this call, with the Chang’E 5 mission bringing samples back from the Moon on December 16, 2020—the first lunar samples returned since Luna. NASA’s recently launched Artemis program aims to send humans back to the Moon and establish a pit-stop on the way to Mars. Furthermore, sealed Apollo samples await opening and first analysis through the Apollo Next Generation Sample Analysis initiative.

What can be learned from analyzing 50-year-old lunar samples? Even samples that have been extensively studied still hold secrets to be unveiled by modern analytical techniques, making “old” new once more.

## Discovering new pieces to an ancient puzzle

New research^5^ led by William Nelson of the University of Hawai’i at Mānoa adds value to one of the most well-studied samples in the Apollo collection: lunar troctolite 76535. Lunar troctolites are a rock type within a group of ultramafic, plutonic rocks from the Moon known as the “Mg-suite.”

The Mg-suite samples provide important insights into planetary differentiation. The source region for the Mg-suite is believed to be chemically tied to the first mantle products crystallizing from a global magma ocean. However, the Mg-suite rocks also contain elevated concentrations of incompatible trace elements thought to be a signature of late-stage magma ocean products, i.e., a “KREEP” component enriched in potassium (K), rare earth elements (REE), and phosphorus (P). This conjunction of primary and late-stage magma ocean products characterizes the complex history that lunar scientists are still working to unravel.

Having intruded into the primary lunar crust, Mg-suite magmas represent the first stages of secondary crust building on the Moon. Following this, Nelson and colleagues point out a puzzling observation: previous cooling rate estimates for Mg-suite samples indicate that the rocks cooled slowly within the crust for over 100 million years^6^. However, this slow cooling rate requires an age for Mg-suite magmas (4421 ± 11 Ma) that pre-dates the formation of the primary lunar crust (4383 ± 17 Ma^7,8^). Given that the primary crust must have formed prior to the magmas that later intruded it, the thermal evolution of the Mg-suite rocks warrants re-investigation.

Using high-resolution analytical techniques, Nelson and colleagues mapped the concentration of the trace element phosphorus within troctolite 76535. Phosphorus diffusion patterns preserved in olivine grains were consistent with a rapid cooling history at high temperatures. The observed diffusion patterns could only be reproduced with a two-stage model involving rapid cooling at high temperatures and slow cooling at lower temperatures. In contrast to the previously proposed 100-million-year cooling duration, this result supports initial rapid cooling of Mg-suite magmas within the lunar crust.

These findings motivate the re-examination of additional olivine-bearing Mg-suite samples in order to validate the cooling history determined for 76535 and to investigate any regional variations between landing sites. More importantly, Nelson and colleagues demonstrate the worth of reanalyzing 50-year-old samples and how quickly new data can reshape our understanding of planetary evolution.

## Mid-century moonwalks, modern measurements

**Fig. 1:**
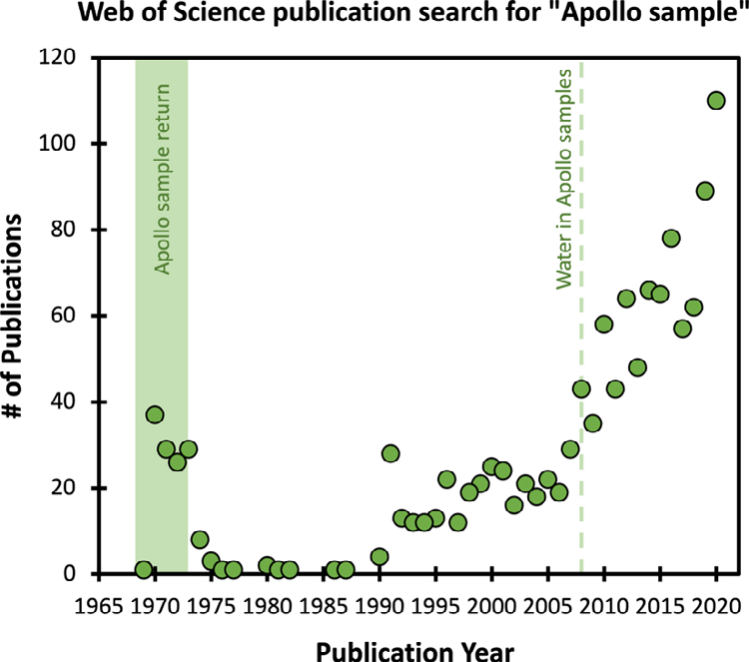
Results from a Web of Science search for “Apollo sample” in works published prior to 2021. As would be expected, a spike in publications exists during the initial Apollo sample return in 1969–1972. A marked increase in publications is observed beginning in the 1990s, with significant growth in publications after 2005. Along with the orbital remote sensing missions at the turn of the century, the discovery of water in lunar samples has generated a sample science resurgence that continues to the present.

Technological advancements post-Apollo Era have facilitated breakthroughs in lunar science. For instance, the discovery of water within lunar volcanic glasses^3^ and apatites^4^ uprooted over 40 years of consensus that the Moon was nominally anhydrous. This prompted a paradigm shift and the total number of publications featuring lunar sample science has been increasing ever since (Fig. 1). Further, the ability to analyze volatiles in lunar samples half a century after their return to Earth demonstrates the long-term value of sample return missions and the importance of proper sample curation.

Modern techniques for volatile element measurement include in situ analyses, which, compared to bulk sample analysis, have lower detection limits and decreased potential for terrestrial contamination. Specifically, advances in secondary ion mass spectrometry have improved detection limits by two orders of magnitude and enabled detection of water within lunar samples that is equivalent to concentrations observed in mid-ocean ridge basalts on Earth. Following this discovery, Moon formation models must now account for the preservation of volatile elements during the high-energy giant-impact event.

Previously unidentified differences in the isotopic compositions of lunar rocks have reinvigorated debates and prompted new questions in lunar science. For example, the analytical precision of iron isotopic measurements by multicollector-inductively coupled plasma mass spectrometer has improved by an order of magnitude in the past decade. In the early 2000s, analyses of the low-Ti and high-Ti lunar mare basalts yielded identical iron isotopic compositions; however, recent re-analysis of these samples has revealed measurable iron isotopic fractionation between the two rock types^9^. Further, the bulk isotopic compositions of the Earth and Moon are now recognized as distinct for certain elements (e.g., potassium^10^). These observations warrant revision of existing hypotheses related to lunar volcanism and the Moon-forming impact, as new hypotheses must identify processes that simultaneously produce each of the observed compositional variations.

Just as re-investigation of lunar samples can lead to new insights, in some cases, modern studies breathe life into old hypotheses. For instance, Prissel and Gross^11^ contributed to the tale of lunar troctolites by combining new data from lunar meteorites, orbital remote sensing, and experimental petrology. They concluded that the Mg-suite primary source was KREEP-free and proposed a model that involves low-degree partial melting of the lunar mantle followed by extensive fractional crystallization. This redefinition of the canonical Mg-suite differentiation trend was originally proposed by a study^12^ of lunar pyroxene compositions. Now, samples collected from the Procellarum KREEP Terrane by the Chang’E 5 mission are providing further evidence for KREEP-free partial melting of the lunar interior^13^. These independent approaches converge on a similar conclusion and exemplify how, after decades of work, lunar exploration and research will continue to provide new pieces to the ancient puzzle of lunar volcanism.

## To the Moon and back

Deciphering the history of our ancient Moon requires a synergy of sample science, remote sensing, and experimentation. Exposures of ancient mantle material identified at the South Pole-Aitken impact basin^14^ provide a tantalizing target for future sample-return missions and, when combined with the relatively young volcanic material returned by Chang’E 5^15^, would provide critical insight into the evolution of the lunar mantle. Collecting additional samples from previously unexplored regions helps minimize any sampling bias that may exist in our lunar collection; after Apollo 11 discovered high-Ti mare basalts, Apollo 12 sampled younger, low-Ti mare basalts, immediately enhancing our knowledge of the sources and timing of lunar volcanism. Lingering questions regarding the initiation of and heat sources for lunar volcanism could be answered by further sampling outside of the unique KREEP-rich terrane on the lunar nearside. Although samples from the lunar farside have been brought to Earth as meteorites, these lack the geologic context offered by sample-return missions. Now, perhaps more than ever, we recognize the value not only of exploring our solar system but also returning home.
