# Author Correction: A lunar sample renaissance

**DOI:** 10.1038/s41467-024-45993-7

**Published:** 2024-02-21

**Authors:** Tabb C. Prissel, Kelsey B. Prissel

**Affiliations:** Jacobs-JETS, Astromaterials Research and Exploration Science Division, NASA Johnson Space Center, Houston, TX USA

**Keywords:** Petrology, Planetary science, Geochemistry

Correction to: *Nature Communications* 10.1038/s41467-021-27296-3, published online 14 December 2021

The original version of this Article contained an error in the first paragraph, which incorrectly read ‘and the Soviet Union’s Luna (1976) sample return missions’ The correct version states ‘(1970-1976)’ in place of ‘(1976)’. This has been corrected in both the PDF and HTML versions of the Article.

